# Impact of Elexacaftor–Tezacaftor–Ivacaftor on Muscle Composition in Cystic Fibrosis: An AI-Assisted Chest CT-Based Body Composition Analysis

**DOI:** 10.3390/medsci13040284

**Published:** 2025-11-26

**Authors:** Matthias Welsner, Florian Stehling, Wolfgang Gruber, Dirk Westhölter, Sivagurunathan Sutharsan, Christian Taube, Erik Büscher, Felix Nensa, Sebastian Zensen, Lale Umutlu, Michael Forsting, Johannes Haubold, Luca Salhöfer, Mathias Holtkamp, Judith Kohnke, Rene Hosch, Marcel Opitz

**Affiliations:** 1 Department of Pulmonary Medicine, University Hospital Essen—Ruhrlandklinik, Adult Cystic Fibrosis Center, University of Duisburg-Essen, 45239 Essen, Germany; 2 Pediatric Pulmonology and Sleep Medicine, Cystic Fibrosis Center, Children’s Hospital, University of Duisburg-Essen, 45147 Essen, Germany; 3 Institute for Artificial Intelligence in Medicine, University Medicine Essen, 45131 Essen, Germany; 4 Institute of Diagnostic and Interventional Radiology and Neuroradiology, University Hospital Essen, University of Duisburg-Essen, 45147 Essen, Germany

**Keywords:** cystic fibrosis, adults, chest-computed tomography, body composition, artificial intelligence, sarcopenia, myosteatosis

## Abstract

Background: This study aimed to investigate longitudinal changes in muscle mass, quality, and composition (sarcopenia and myosteatosis) in adult people with cystic fibrosis (pwCF) using artificial intelligence (AI)-assisted body composition analysis (BCA) with chest computed tomography (CT) at the T12 level and to examine the influence of CFTR modulator therapy with elexacaftor/tezacaftor/ivacaftor (ETI). Methods: A retrospective observational study was conducted on 102 adult pwCF (42 females (41%), mean age 33.9 ± 11.1 years) who underwent routine chest CT scans with a minimum of six months between scans. PwCF were categorized into ETI and no ETI groups. AI-assisted BCA was performed on chest CT images at the T12 level to measure skeletal muscle area (SMA), inter- and intramuscular adipose tissue (IMAT), and low-attenuation muscle area (LAMA). IMAT/SMA ratio and height- and weight-related skeletal muscle indices (SMI) were calculated. Results: The ETI group showed a significant increase in SMA over time (*p* < 0.001), whereas the IMAT, LAMA, and IMAT/SMA ratio increased in both groups (all *p* < 0.05). SMI showed alterations only in the ETI group, with an increase in SMA/m^2^ (*p* < 0.001) and a decrease in SMA/kg (*p* = 0.003) and SMA/BMI (*p* = 0.006). Sex-specific analysis showed that SMA and myosteatosis increased regardless of sex (all *p* < 0.05). Weight-adjusted SMI decreased only in females receiving ETI therapy (*p* < 0.05). Conclusions: Adult pwCF, particularly those undergoing ETI therapy, experience significant changes in body composition, including increased muscle mass and myosteatosis. Trends in the development of sarcopenic obesity have been observed, particularly in female pwCF. These findings emphasize the importance of comprehensive body composition assessments and targeted interventions in pwCF treated with ETI to optimize muscle mass and quality while managing adipose tissue accumulation.

## 1. Introduction

Cystic fibrosis (CF) is a genetic disorder caused by mutations in the Cystic Fibrosis Transmembrane Conductance Regulator (CFTR) protein, an anion channel in the apical membrane of epithelial cells that primarily affects the respiratory and digestive systems; however, its impact extends to muscle health [1]. Sarcopenia, characterized by the loss of skeletal muscle mass and function, is a significant concern in people with CF (pwCF). The association between CF and sarcopenia is complex and multifaceted. As CF progresses, it initiates a cascade of physiological changes that directly and indirectly affect muscle health. Chronic inflammation associated with CF can lead to increased protein breakdown and impaired muscle protein synthesis. Additionally, malnutrition due to pancreatic insufficiency and malabsorption compromises muscle maintenance and growth [2]. Reduced physical activity, which is frequently a consequence of respiratory limitations, further exacerbates muscle deconditioning and atrophy [3]. Moreover, aberrant CFTR function leads to inadequate regulation of calcium homeostasis, thereby affecting muscle depolarization. Dysregulation of these channels may cause muscle mass loss and weakness [4,5]. Consequently, muscle wasting can reduce lung function, diminish exercise capacity, and lower the overall quality of life [2].

In sarcopenia research, muscle quality is considered more crucial than muscle mass [6]. The functionality and condition of the muscles play a more important role than the quantity of muscle tissue, underscoring the importance of maintaining muscle quality rather than merely increasing muscle mass to prevent or manage sarcopenia.

Distinct from sarcopenia, myosteatosis refers to pathological fat infiltration in muscles and is associated with muscle fiber disarrangement, disrupted muscle contractility, and weakened mechanical action, resulting in decreased muscle quality and function [7,8]. Myosteatosis comprises three components: (1) intramyocellular lipids (IMCL); (2) intramuscular adipose tissue (IntraMAT), extracellular fat within a given muscle; and (3) intermuscular adipose tissue, extracellular fat located beneath the fascia, and between muscle groups [9]. The etiology of myosteatosis is multifactorial, including impaired mitochondrial oxidative phosphorylation, diminished lipid oxidation in muscles, and age-related differentiation of muscle stem cells into adipocytes [10]. This condition can occur independently or in conjunction with sarcopenia and is associated with various adverse health outcomes [11,12].

The European Working Group on Sarcopenia in Older People (EWGSOP2) defines sarcopenia through a standardized, stepwise algorithm based on three criteria: low muscle mass, low muscle strength, and low physical performance [13]. Muscle mass is assessed using dual-energy X-ray absorptiometry (DXA), the reference method, or bioelectrical impedance analysis (BIA) as a feasible alternative. Muscle strength is evaluated via handgrip dynamometry, and physical performance is determined by the 4-m usual gait speed test. Sarcopenia is diagnosed when low muscle strength and low physical performance are present, with low muscle mass confirming the diagnosis. This integrated approach ensures accurate identification of clinically relevant sarcopenia, facilitating early intervention and improved patient outcomes.

Computed tomography (CT) has become an increasingly popular alternative imaging method for assessing myosteatosis and sarcopenia [14]. This technique offers high-resolution cross-sectional images, enabling precise evaluation of muscle mass by measuring the skeletal muscle area (SMA). Moreover, CT enables the detection of myosteatosis not only through direct quantification of intermuscular and intramuscular adipose tissue (IMAT) but also through indirect estimation of IntraMAT and IMCL via the measurement of skeletal muscle radiodensity (SMD), expressed in Hounsfield Units (HU). A lower HU value indicates decreased radiodensity, corresponding to elevated IntraMAT and IMCL levels. A recent investigation demonstrated that the assessment of muscle mass and quality using chest CT scans at the T12 level is efficacious for diagnosing sarcopenia and myosteatosis [15].

Despite extensive research over the past decade documenting muscle weakness and dysfunction in pwCF, there is limited knowledge regarding the prevalence and potential clinical significance of myosteatosis in this population.

In recent years, CFTR modulator therapy has transformed the standard of care for CF, signifying a major advancement in the therapeutic landscape [16]. These modulators target the underlying genetic defect in CF by enhancing CFTR protein function. This approach represents a departure from traditional symptom-based treatments and offers a more targeted and potentially effective intervention.

The implementation of CFTR modulators, particularly triple therapy comprising elexacaftor, tezacaftor, and ivacaftor (ETI), in clinical practice has markedly enhanced outcomes in pwCF. These improvements are reflected in better lung function, reduced pulmonary exacerbations, higher quality of life, and significant improvements in nutritional status [17,18]. However, recent studies have raised concerns regarding the composition of the resulting weight gain. Evidence suggests that much of the increased weight may be derived from fat mass accumulation, including IMAT, as opposed to a balanced gain in both fat and fat-free mass [19,20,21]. This trend shifts pwCF towards overweight and obesity, elevating the risk of cardiovascular complications [22,23].

The primary objective of this study was to investigate longitudinal alterations in muscle mass, quality, and composition (i.e., sarcopenia and myosteatosis) in adult pwCF using a fully automated artificial intelligence (AI)-assisted body composition analysis of chest CT scans at the T12 level. The secondary aim was to explore the effects of CFTR modulator therapy with ETI on these parameters. Using AI-assisted body composition analysis, we sought to elucidate the physiological changes that occurred following the initiation of this therapy.

## 2. Material and Methods

### 2.1. Study Design and Participants

This retrospective observational study was conducted at the Adult Cystic Fibrosis Center of the Ruhrlandklinik Essen, Germany. The manuscript was prepared following the Strengthening the Reporting of Observational Studies in Epidemiology (STROBE) guidelines [24]. A completed STROBE checklist is provided in the Supplementary Material.

The study population comprised individuals aged ≥ 18 years with a confirmed diagnosis of CF. Routine chest CT scans, with a minimum of six months between scans, were identified by reviewing electronic medical records. Relevant demographic and disease-specific information, including lung function test results and biometric measurements, were extracted from digital files. For each patient, the data point (T0: baseline and T1: follow-up) closest to the chest CT was selected for analysis. PwCF were subsequently categorized into two groups: those receiving CFTR modulator therapy with ETI (ETI group) and those not receiving therapy (no ETI group, control group).

The study was approved by the Institutional Review Board (IRB) of the University Hospital Essen (24-11977-BO, date of approval: 26 June 2024) and followed the ethical principles of the Declaration of Helsinki for medical research involving human subjects.

### 2.2. CT Image Acquisition and Body Composition Analysis

Body composition was evaluated by analyzing chest CT images acquired as part of routine clinical practices. A 64-detector row single-source CT scanner (Somatom Definition AS; Siemens Healthineers, Erlangen, Germany) was used for all chest CT examinations. The scanner parameters included a 300 ms gantry rotation time, 64 × 0.6 mm collimation, 1.0 mm slice thickness, 25 mAs tube current-time product, and 120 kV tube voltage. Radiation protection was enhanced by implementing automatic tube current modulation (CARE Dose4D) and tube voltage selection (CARE kV algorithm). All CT scans were performed without contrast enhancement.

**Figure 1:**
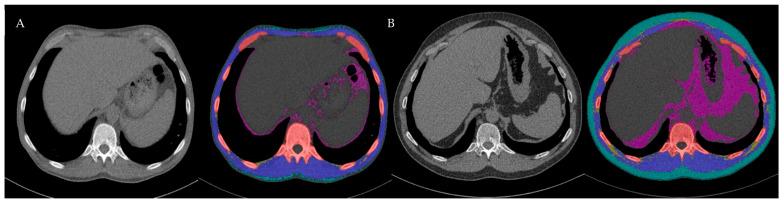
Cross-sectional CT images illustrating AI-derived body composition analysis (BCA) at the T12 level and its changes over time. Bone (red), muscle (blue), visceral adipose tissue (purple), subcutaneous adipose tissue (turquoise), and IMAT (yellow). (**A**) Pre ETI: SMA 92.5 cm^2^, IMAT 4.0 cm^2^, IMAT/SMA 4.3%; (**B**) post-ETI: SMA 120.2 cm^2^, IMAT 19.6 cm^2^, IMAT/SMA 16.3%. ETI, elexacaftor, tezacaftor, ivacaftor; SMA, skeletal muscle area; IMAT, intermuscular and intramuscular adipose tissue; LAMA, low-attenuation muscle area.

PwCF were scanned in an inspiratory breath-hold and head-first supine position with their arms elevated. For further image processing, reconstruction in the soft tissue window B31F with a 1 mm slice thickness was used to perform body composition analysis (BCA). BCA is a component of an open-source body organ analysis (BOA) platform [25,26]. This technique combines various segmentation methods and prioritizes an efficient workflow by incorporating DICOM node functionality. It also enables highly precise automatic tissue segmentation independent of dose-related scan parameters [27]. In addition to tissue-specific segmentations, the model computes volumetric and density-dependent features, providing a comprehensive three-dimensional characterization of the body composition. To complement the volumetric analysis, the BOA model performs cross-sectional segmentation. In this study, only cross-sectional segmentation from the median slice of the T12 vertebral body was extracted to ensure a consistent anatomical reference point for comparability between patients (Figure 1).

In the postprocessing step, the cross-sectional area of the skeletal muscle (SMA, cm^2^) was measured at the T12 level. The SMA includes the erector spinae, latissimus dorsi, intercostal, rectus abdominis, and internal and external oblique muscles.

Furthermore, measurements were conducted for low-attenuation muscle area (LAMA) and intermuscular and intramuscular adipose tissue (IMAT). IMAT was identified within the segmented muscles by applying a filter in the range of −30 to −190 HU. Similarly, LAMA was determined within the segmented muscle regions using a filter range of −29 to 29 HU. The results are expressed as absolute values (cm^2^) and percentages of SMA (%IMAT = IMAT/(SMA + IMAT) × 100; %LAMA = (LAMA/SMA) × 100) [28]. The IMAT/SMA ratio was calculated as a percentage (IMAT/SMA × 100). Additionally, skeletal muscle indices (SMI) were calculated by normalizing SMA to height, weight, and BMI.

### 2.3. Statistical Analysis

Statistical analyses were performed using SPSS version 29 (SPSS Inc., Chicago, IL, USA). The results are expressed as mean ± standard deviation and 95% confidence intervals (CI). The Shapiro–Wilk test was used to evaluate the normality of the distribution. For intergroup comparisons, independent t-tests were applied to normally distributed variables, whereas the Mann–Whitney U test was used for non-normally distributed data. Continuous data were analyzed using the Wilcoxon test or paired t-test, as appropriate. Chi-square or Fisher’s exact tests were used to analyze categorical variables. Statistical significance was set at *p* < 0.05.

## 3. Results

### 3.1. Patient Characteristics

The final study cohort comprised 102 adult pwCF, of whom 42 (41%) were female (Table 1). The mean age was 33.9 ± 11.1 years, with a mean ppFEV1 of 46.4 ± 21.4 and a mean body mass index (BMI) of 21.1 ± 3.6 kg/m^2^ at the time of the first chest CT scan. CT scans were performed between November 2017 and December 2024. Most participants (44%) were homozygous for F508del (44%). Among the pwCF, 93 (91%) had exocrine pancreatic insufficiency, and 21 (21%) had a diagnosis of CF-related diabetes mellitus (Table 1).

ETI treatment was initiated in 80 of the 102 pwCF (78%) during the observation period (ETI group). The interval between the two chest CT examinations was 1024.3 ± 401.3 days in the ETI group and 1179.7 ± 378.3 days in the noETI group (*p* = 0.107). The duration between the initiation of ETI therapy and follow-up CT was 848.9 ± 312.6 days. No statistically significant differences were observed in terms of age (*p* = 0.918), ppFEV1 (*p* = 0.082), or BMI (*p* = 0.125) between the groups at the time of the first CT scan (T0).

For all pwCF, a significant improvement (*p* < 0.001) was observed in mean ppFEV1, which increased from 46.4 ± 21.4 at the initial assessment (T0) to 52.8 ± 22.1 during follow-up (T1). BMI demonstrated a substantial increase (*p* < 0.001), changing from 21.1 ± 3.6 to 22.5 ± 3.7 (*p* < 0.001). In the subgroup analysis, statistically significant improvements in ppFEV1 (8.1 ± 10.0; *p* < 0.001) and BMI (1.7 ± 1.9; *p* < 0.001) were observed only in the ETI group (Table 2).

### 3.2. Course of Body Composition at T12 Level Under ETI

The results presented in Table 3 provide a comprehensive overview of the automated muscle assessments at the T12 vertebral level, revealing significant changes in muscle composition over time. Overall, longitudinal data analysis demonstrated substantial increases across all measured muscle parameters (SMA, IMAT, %IMAT, LAMA, and %LAMA; all *p* < 0.001), including IMAT/SMA ratio (*p* < 0.001). Further subgroup analysis indicated that only the ETI group experienced a significant increase in SMA (*p* < 0.001). Both the ETI and no-ETI groups exhibited significant increases in IMAT, %IMAT, LAMA, and %LAMA (all *p* < 0.01) over time. The associated SMI demonstrated significant alterations exclusively in the ETI group (all *p* < 0.01) but remained nearly unchanged in the no-ETI group (all *p* > 0.05).

### 3.3. Sex-Specific Changes and Differences During Treatment with ETI

Table 4 displays the sex-specific changes and differences in clinical outcomes and body composition of the ETI group. The interval between the onset of ETI and subsequent CT scan was 897.4 ± 302.3 days for men and 792.5 ± 318.9 days for women (*p* = 0.135). Analysis by sex revealed a notable response to ETI therapy in both males and females, with significant improvements in lung function and BMI (*p* < 0.001 for both). At the T12 level, the cross-sectional measurement of skeletal muscle showed an increase in SMA in both sexes (*p* = 0.002 for each). Significant increases were observed in both the absolute and relative measures of IMAT (all *p* < 0.001) and in the measured absolute and percentage values of LAMA. The IMAT/SMA ratio significantly increased in both men and women (*p* < 0.001). SMA/m^2^ increased in both male (*p* < 0.001) and female (*p* = 0.004). However, the weight-related SMI measures of SMA/kg and SMA/BMI showed a significant decrease only in females (SMA/kg, *p* = 0.015; SMA/BMI, *p* = 0.017).

## 4. Discussion

This long-term investigation, utilizing fully automated AI-assisted analysis of chest CT scans at the T12 level, revealed significant alterations in muscle mass, quality, and composition over time in adult pwCF.

Our findings indicate an increase in SMA exclusively among pwCF using ETI over the observed period. Irrespective of ETI usage, both groups exhibited an increase in myosteatosis (IMAT and LAMA) and an increase in the IMAT/SMA ratio, indicating a higher proportion of fat relative to the muscle area. Within the ETI group, alterations in SMI were noted, with an increase in SMI (SMA/m^2^), indicating a growth in muscle mass relative to height. Conversely, there was a slight decrease in SMA/kg and SMA/BMI, suggesting that the increase in muscle mass did not correspond to the overall weight or BMI increase. This discrepancy suggests a potential development of sarcopenic obesity. Gender-specific analysis of pwCF on ETI therapy revealed increases in markers of myosteatosis and SMA in both sexes. Weight-adjusted SMI decreased only in females. These findings emphasize the importance of comprehensive body composition assessment in pwCF undergoing ETI treatment and underscore the need to identify targeted interventions to optimize muscle mass and quality while managing adipose tissue accumulation in this population.

When comparing our results with age-adjusted and sex-related normative data at the T12 level, pwCF demonstrated lower values for SMA and SMI (cm^2^/m^2^), indicating reduced muscle mass compared to healthy individuals [29]. When the proposed cutoff values at T12 were applied to define sarcopenia (female: SMA 56.2 cm^2^, SMI 20.8 cm^2^/m^2^; male: SMA 92.3 cm^2^, SMI 28.8 cm^2^/m^2^), no such diagnosis could be established in the study population. However, the applicability of the cutoffs specified for healthy adults to people with chronic illness or their utility in defining sarcopenia in cystic fibrosis remains uncertain. Despite potential increases in SMA and SMI during ETI treatment, age- and sex-specific normal values remained unattainable.

In CF, comparison with other studies is challenging because of substantial variability in the disease. In their investigation, Jennerich et al. demonstrated significantly lower SMA and SMI values at T12 in pwCF pre-transplant compared to our cohort, albeit in significantly more compromised pwCF [30]. Conversely, the Navas-Moreno study observed slightly higher values in a younger and better-preserved pwCF cohort than those in our study population [20]. The outcomes of our investigation lie between those of the two aforementioned studies.

In terms of myosteatosis, a comparative analysis of normative data from a young population revealed notably reduced values, in contrast to our study group, specifically regarding IMAT [cm^2^] and IMAT/SMA ratio at the T12 vertebral level [15]. This finding suggests the potential for myosteatosis in adult pwCF. However, it is crucial to acknowledge that the reference data originated from an Asian comparative group, which limits the broader applicability of these results. To the best of our knowledge, there are no established normative values for IMAT and related measurements at the T12 level in the Caucasian population. Furthermore, there is a lack of consensus regarding a standardized definition of this condition [11]. The absence of a standardized definition poses significant challenges for research and clinical practice. This impedes the comparability of studies and may result in inconsistencies in the diagnosis and treatment of myosteatosis. Establishing a consensus definition and standardized assessment methodologies for myosteatosis is essential for advancing our understanding of this condition and its implications for cardiometabolic health and other clinical outcomes.

Our findings revealed a significant increase in IMAT and LAMA, which occurred independently of ETI in both female and male pwCF. This observation aligns with previous studies that established a correlation between aging and increased myosteatosis in healthy individuals [9,31]. However, our study demonstrated that the increase in IMAT was approximately twice as pronounced in the ETI group compared to the non-ETI group. This difference suggests the presence of two distinct effects: one related to ETI and the other associated with the natural aging process. The implications of these findings are substantial, as they indicate that ETI may accelerate or exacerbate adipose tissue accumulation within muscle structures.

Given that female pwCF exhibit a significant decrease in weight-adjusted SMI compared with their male counterparts, female pwCF appear to be at a higher risk of developing sarcopenic obesity.

### 4.1. Exercise and Body Composition

Physical activity is the most important intervention for the prophylaxis and treatment of sarcopenia and myosteatosis [8,32].

In healthy adults, research has shown that physical activity and exercise, particularly resistance training and high-intensity interval training (HIIT), can improve body composition by reducing body fat percentage, body fat mass, and visceral fat, while increasing fat-free mass [33,34]. Although this study did not specifically address pwCF, it suggests that resistance training and HIIT may be viable approaches for improving body composition in pwCF. This assumption is supported by a cross-sectional study by Scully et al., who examined the relationship between body composition, dietary intake, physical activity, and pulmonary status in adolescents and adults with CF and showed that higher levels of physical activity were associated with greater lean muscle mass in the upper and lower extremities and lower body fat mass [35].

Transferring these findings to our data, we hypothesized that the observed increase in SMA among pwCF treated with ETI was a consequence of elevated physical activity levels. This elevation may be attributed to the improved pulmonary function and ameliorated respiratory symptoms experienced by pwCF using ETI. Enhanced respiratory capacity may facilitate greater engagement in daily activities and exercise regimens, thereby contributing to the observed increase in SMA. Nevertheless, it must be acknowledged that there is a lack of randomized controlled trials in pwCF, particularly to assess the effects of exercise and physical activity on body composition and physical activity levels, highlighting a significant research gap [36]. Additional studies are warranted to elucidate the relationship among exercise, physical activity, and body composition, particularly their effects on sarcopenia and myosteatosis.

### 4.2. Nutritional Intake/Interventions

In addition to physical activity and exercise, nutritional interventions play a crucial role in addressing weight gain by implementing a range of targeted dietary modifications and strategic approaches.

For numerous years, the predominant care protocol for pwCF has emphasized a diet high in fat and calories, acknowledging the critical role of nutritional status in respiratory function and overall survival [37]. Dietary approaches are comprehensive strategies designed to address the unique nutritional challenges associated with this genetic disorder. This approach aims to counteract the increased energy expenditure and nutrient malabsorption commonly observed in pwCF, primarily due to pancreatic insufficiency and chronic lung and gut inflammation. These physiological complications often result in higher caloric requirements and difficulty absorbing essential nutrients from food.

Following nutritional requirements, pwCF typically consume more calories than their healthy counterparts, and their diet is characterized by increased fat intake and higher glycemic foods [35].

The widespread use of CFTR modulators has dramatically changed the nutritional status of pwCF, leading to a higher prevalence of overweight and obesity [38]. Notably, despite an increase in BMI, a significant decrease in energy intake was observed in pwCF undergoing ETI therapy [39,40]. This finding contradicts the initial hypothesis that an increased BMI associated with ETI therapy is solely attributable to continued CF-specific dietary recommendations. The observed decrease in energy intake, concurrent with weight gain, suggests the involvement of alternative mechanisms. For instance, ETI may enhance nutrient absorption, reduce energy expenditure, and modify metabolic processes in pwCF. These potential mechanisms warrant further investigation through longitudinal studies that combine metabolic assessments and detailed dietary analyses. Such research could provide valuable insights into the metabolic changes induced by ETI therapy and inform the development of tailored nutritional guidelines for the treatment of pwCF. Additionally, exploring the role of gut microbiota in nutrient absorption and metabolism in the context of ETI therapy may offer new perspectives on weight-management strategies for this patient population.

The combination of targeted nutritional interventions, regular physical activity, and exercise creates a synergistic effect that enhances muscle strength and metabolism more effectively than either approach alone. This comprehensive strategy not only addresses the immediate concerns of weight gain, sarcopenia, and myosteatosis but also contributes to long-term health benefits, including improved body composition, enhanced functional capacity, and reduced risk of chronic diseases associated with excess body fat and muscle loss. The importance of combined approaches to healthcare has become even more evident in the context of increasing life expectancy and the expected increase in disease-specific and non-disease-specific age-related comorbidities, as in pwCF [41]. Integrating preventive measures, early detection strategies, and comprehensive treatment plans can help to address the complex health needs of adult pwCF.

Our study has several important limitations. First, although our cohort included a relatively large number of adult pwCF, the use of data from a single center limits its generalizability. Second, CT-based BCA was restricted to chest CT scans because abdominal and full-body scans were not available. As body composition can vary across different regions, this may limit the generalizability of our findings. However, previous studies utilizing both manual and automated segmentation techniques have shown that chest CT scans can serve as a reliable proxy for whole-body composition assessment [42]. Finally, our analysis was limited by the absence of functional performance measures (e.g., gait speed) and dynamometry (e.g., handgrip strength), as well as DXA or BIA, which are essential components of the EWGSOP2 criteria for diagnosing sarcopenia [13].

## 5. Conclusions

This retrospective longitudinal study, using AI-assisted analysis of chest CT scans at the T12 level, revealed significant changes in muscle mass, quality, and composition among adult pwCF, especially those receiving CFTR modulator therapy with ETI. The findings suggest a trend towards sarcopenic obesity, notably in females using ETI. These results underscore the necessity of refined weight management and nutritional guidance for pwCF undergoing ETI therapy. Alterations in body composition emphasize the importance of integrating targeted nutritional interventions with regular physical activity and exercise to maintain optimal muscle mass and quality. As the life expectancy of pwCF continues to rise, addressing these changes in body composition has become increasingly crucial for managing disease-specific and age-related comorbidities.

## Figures and Tables

**Table 1:** Patient demographics and clinical characteristics at baseline (T0).

Characteristics	All (n = 102)	ETI (n = 80)	No-ETI (n = 22)	*p*-Value ETI vs. No-ETI
Age, years	33.9 ± 11.1 [31.7–36.0]	33.9 ± 11.2 [31.4–36.4]	33.6 ± 11.1 [28.7–38.6]	0.918
Female sex, n (%)	42 (41)	37 (46)	5 (23)	0.047
Genotype, n (%)				<0.001
F508del homozygous	45 (44)	45 (56)	0	
F508del heterozygous	32 (31)	32 (40)	0	
Other	25 (25)	3 (4)	22 (100)	
Pancreatic insufficiency, n (%)	93 (91)	75 (94)	18 (82)	0.035
Cystic fibrosis-related diabetes, n (%)	21 (21)	18 (23)	3 (14)	0.003
Pseudomonas aeruginosa	52 (51)	46 (58)	6 (27)	0.016
Body mass index [kg/m^2^]				0.433
Underweight (<18.5)	26 (25)	22 (28)	4 (18)	
Normal/healthy weigt (18.5–24.9)	61 (60)	48 (60)	13 (59)	
Overweight/Obese (>25)	15 (15)	10 (12)	5 (23)	
ppFEV1				0.125
Normal (>90)	6 (6)	4 (5)	2 (9)	
Mild obstruction (70–90)	11 (11)	7 (9)	4 (18)	
Moderate obstruction (40.70)	38 (37)	30 (38)	8 (36)	
Severe obstruction (<40)	47 (46)	39 (49)	8 (36)	

Values are expressed as mean ± standard deviation or number of patients (%). Brackets indicate the 95% confidence intervals (CI). ETI, elexacaftor, tezacaftor, ivacaftor; ppFEV1, percent predicted Forced Expiratory Volume in one second.

**Table 2:** Longitudinal Comparison of Clinical Characteristics Between the ETI and No-ETI Groups.

Characteristics	All (n = 102)	ETI Group (n = 80)	No ETI Group (n = 22)	*p*-Value ETI vs. No ETI
Time between T0 and T1 [days]	1057.8 ± 399.8 [979.3–1136.3]	1024.3 ± 401.3 [935.0–1113.6]	1179.7 ± 378.3 [1012.0–1347.5]	0.107
ppFEV1				
T0	46.4 ± 21.4 [42.2–50.6]	44.3 ± 20.1 [39.8–48.8]	54.0 ± 24.5 [43.1–64.8]	0.082
T1	52.8 ± 22.1 [48.5–57.2]	52.5 ± 21.0 [47.8–57.2]	54.1 ± 26.2 [42.5–65.7]	0.756
Mean difference	6.4 ± 10 [4.4–8.4]	8.1 ± 10.0 [5.9–10.4]	0.2 ± 7.4 [−3.1–3.5]	
*p*-value	<0.001	<0.001	0.909	
FEV1 [L]				
T0	1.8 ± 1.0 [1.6–2.0]	1.7 ± 1.0 [1.5–1.9]	2.1 ± 1.0 [1.7–2.6]	0.108
T1	2.0 ± 1.0 [1.8–2.2]	2.0 ± 1.0 [1.8–2.2]	2.1 ± 1.1 [1.6–2.6]	0.789
Mean difference	0.2 ± 0.4 [0.1–0.3]	0.3 ± 0.4 [0.2–0.4]	−0.03 ± 0.3 [−0.2–0.1]	
*p*-value	<0.001	<0.001	0.603	
Body weight [kg]				
T0	62.3 ± 13.5 [59.7–64.9]	61.3 ± 13.0 [58.4–64.2]	65.9 ± 14.9 [59.3–72.5]	0.160
T1	66.4 ± 14.2 [63.6–69.2]	66.4 ± 14.0 [63.3–69.6]	66.3 ± 15.1 [59.7–73.0]	0.890
Mean difference	4.1 ± 6.5 [2.8–5.4]	5.1 ± 6.1 [3.8–6.5]	0.5 ± 6.7 [−2.5–3.4]	
*p*-value	<0.001	<0.001	0.883	
BMI [kg/m^2^]				
T0	21.1 ± 3.6 [20.4–21.8]	20.7 ± 3.2 [20.0–21.4]	22.6 ± 4.9 [20.4–24.7]	0.125
T1	22.5 ± 3.7 [21.7–23.2]	22.4 ± 3.3 [21.6–23.1]	22.9 ± 5.1 [20.6–25.1]	0.974
Mean difference	1.4 ± 2.1 [1.0–1.8]	1.7 ± 1.9 [1.3–2.1]	0.3 ± 2.4 [0.8–1.3]	
*p*-value	<0.001	<0.001	0.917	

Values are expressed as mean ± standard deviation. Brackets indicate the 95% confidence intervals (CI). ETI, elexacaftor, tezacaftor, ivacaftor; ppFEV1, percent predicted forced expiratory volume in 1 s; BMI, body mass index.

**Table 3:** Body composition analysis (BCA) for sarcopenia and muscle quality assessment at the T12 level.

Characteristics	All (n = 102)	ETI Group (n = 80)	No ETI Group (n = 22)	*p*-Value ETI vs. No ETI
SMA [cm^2^]				
T0	90.5 ± 22.6 [86.0–94.9]	89.2 ± 22.2 [84.2–94.1]	95.2 ± 23.9 [84.6–105.7]	0.274
T1	95.1 ± 25.1 [90.2–100]	94.6 ± 24.3 [89.2–100.0]	96.9 ± 28.4 [84.3–109.5]	0.705
Mean difference	4.6 ± 10.5 [2.6–6.7]	5.4 ± 10.3 [3.1–7.7]	1.8 ± 11.1 [−3.2–6.7]	
*p*-value	<0.001	<0.001	0.464	
IMAT [cm^2^]				
T0	7.4 ± 5.5 [6.3–8.5]	7.0 ± 4.6 [6.0–8.0]	8.8 ± 7.9 [5.3–12.3]	0.855
T1	11.2 ± 7.1 [9.8–12.6]	11.3 ± 6.8 [9.7–12.8]	11.0 ± 8.2 [7.3–14.6]	0.472
Mean difference	3.8 ± 4.5 [2.9–4.7]	4.2 ± 0.9 [3.2–5.3]	2.1 ± 4.1 [0.3–4.0]	
*p*-value	<0.001	<0.001	0.025	
%IMAT				
T0	7.2 ± 4.1 [6.4–8.0]	7.1 ± 3.8 [6.2–7.9]	7.6 ± 5.1 [5.3–9.8]	0.611
T1	10.0 ± 4.4 [9.2–10.9]	10.3 ± 4.4 [9.3–11.2]	9.2 ± 4.5 [7.2–11.2]	0.289
Mean difference	2.9 ± 2.9 [2.3–3.4]	3.2 ± 3.0 [2.5–3.9]	1.7 ± 2.2 [0.7–2.6]	
*p*-value	<0.001	<0.001	0.005	
LAMA [cm^2^]				
T0	26.5 ± 10.0 [24.5–28.5]	26.5 ± 9.5 [24.4–28.6]	26.4 ± 12.0 [21.1–31.7]	0.961
T1	31.1 ± 10.3 [29.0–33.1]	31.1 ± 9.9 [28.9–33.3]	30.9 ± 11.7 [25.7–36.1]	0.921
Mean difference	4.6 ± 5.9 [3.4–5.7]	4.6 ± 5.8 [3.3–5.9]	4.5 ± 6.5 [1.6–7.3]	
*p*-value	<0.001	<0.001	0.004	
%LAMA				
T0	29.0 ± 7.1 [27.6–30.3]	29.5 ± 6.6 [28.0–31.0]	27.0 ± 8.6 [23.2–30.9]	0.150
T1	32.4 ± 5.4 [31.4–33.5]	32.7 ± 5.3 [31.6–33.9]	31.3 ± 5.5 [28.8–33.7]	0.258
Mean difference	3.5 ± 5.5 [2.4–4.5]	3.2 ± 5.5 [2.0–4.5]	4.2 ± 5.5 [1.8–6.7]	
*p*-value	<0.001	<0.001	0.002	
IMAT/SMA ratio				
T0	8.0 ± 5.0 [7.0–8.9]	7.8 ± 4.6 [6.8–8.8]	8.5 ± 6.2 [5.8–11.3]	0.977
T1	11.4 ± 5.6 [10.3–12.5]	11.7 ± 5.7 [10.5–13.0]	10.4 ± 5.6 [7.95–13.0]	0.289
Mean difference	3.5 ± 3.7 [2.8–4.2]	3.9 ± 3.8 [3.1–4.8]	1.9 ± 2.8 [0.7–3.1]	
*p*-value	<0.001	<0.001	0.007	
skeletal muscle indices (SMI)				
SMA/height [m^2^]				
T0	30.6 ± 6.4 [29.3–31.8]	30.0 ± 5.8 [28.7–31.3]	32.7 ± 8.1 [29.1–36.3]	0.080
T1	32.1 ± 7.2 [30.7–33.6]	31.8 ± 6.3 [30.4–33.2]	33.4 ± 9.9 [29.0–37.8]	0.368
Mean difference	1.6 ± 3.4 [0.9–2.2]	1.8 ± 3.3 [1.1–2.6]	0.7 ± 3.9 [−1.0–2.4]	
*p*-value	<0.001	<0.001	0.416	
SMA/kg				
T0	1.5 ± 0.2 [1.4–1.5]	1.5 ± 0.2 [1.4–1.5]	1.5 ± 0.2 [1.4–1.5]	0.905
T1	1.4 ± 0.2 [1.4–1.5]	1.4 ± 0.2 [1.4–1.5]	1.5 ± 0.2 [1.4–1.5]	0.488
Mean difference	−0.02 ± 0.1 [−0.05–0]	−0.03 ± 0.1 [−0.1–0.01]	0 ± 0.15 [−0.1–0.1]	
*p*-value	0.016	0.003	0.931	
SMA/BMI [kg/m^2^]				
T0	4.3 ± 0.7 [4.1–4.4]	4.3 ± 0.7 [4.1–4.4]	4.2 ± 0.7 [3.9–4.6]	0.752
T1	4.2 ± 0.7 [4.1–4.3]	4.2 ± 0.7 [4.0–4.4]	4.2 ± 0.7 [3.9–4.6]	0.878
Mean difference	−0.1 ± 0.3 [−0.1–0]	−0.1 ± 0.3 [−0.2–0]	0 ± 0.4 [−0.2–0.2]	
*p*-value	0.032	0.006	0.941	

Values are expressed as the mean ± standard deviation. Brackets indicate the 95% confidence intervals (CI). ETI, elexacaftor, tezacaftor, ivacaftor; BMI, body mass index; IMAT, intermuscular and intramuscular adipose tissue (−30 to −190 HU); LAMA, low-attenuation muscle area (−29 to 29 HU); SMA, skeletal muscle area.

**Table 4:** Functional data and outcomes of body composition analysis (BCA) at the T12 level for males and females undergoing ETI therapy.

Characteristics	Male (n = 43)	Female (n = 37)	*p*-Value Male vs. Female
Time between start ETI and T1 [days]	897.4 ± 302.3 [804.4–990.5]	792.5 ± 318.9 [686.2–898.9]	0.135
Age [years]			
T0	35.8 ± 12.2 [32.1–39.6]	31.7 ± 9.5 [28.5–34.9]	0.096
T1	38.7 ± 12.2 [35.0–42.5]	34.4 ± 9.6 [31.2–37.6]	0.087
Mean difference	2.9 ± 1.1 [2.6–3.3]	2.8 ± 1.3 [2.3–3.2]	
*p*-value	<0.001	<0.001	
ppFEV1			
T0	48.9 ± 22.5 [42.0–55.9]	38.8 ± 15.3 [33.7–44.0]	0.056
T1	57.0 ± 24.0 [49.6–64.4]	47.1 ± 15.6 [41.8–52.4]	0.100
Mean difference	8.1 ± 10.9 [4.7–11.4]	8.3 ± 8.8 [5.3–11.2]	
*p*-value	<0.001	<0.001	
Body weight [kg]			
T0	68.1 ± 11.9 [64.4–71.7]	53.5 ± 9.2 [50.4–56.5]	<0.001
T1	73.6 ± 12.9 [69.6–77.5]	58.2 ± 10.4 [54.7–61.7]	<0.001
Mean difference	5.5 ± 7.1 [3.3–7.7]	4.7 ± 4.8 [3.1–6.4]	
*p*-value	<0.001	<0.001	
BMI [kg/m^2^]			
T0	21.5 ± 3.0 [20.6–22.5]	19.7 ± 3.1 [18.7–20.7]	0.009
T1	23.2 ± 3.2 [22.2–24.2]	21.4 ± 3.2 [20.4–22.5]	0.016
Mean difference	1.7 ± 2.1 [1.0–2.3]	1.7 ± 1.7 [1.1–2.3]	
*p*-value	<0.001	<0.001	
SMA [cm^2^]			
T0	103.6 ± 19.0 [97.7–109.4]	72.4 ± 11.3 [68.6–76.2]	<0.001
T1	109.7 ± 20.9 [103.3–116.1]	77.1 ± 13.9 [72.4–81.7]	<0.001
Mean difference	6.1 ± 12.0 [2.5–9.8]	4.6 ± 8.2 [1.9–7.4]	
*p*-value	0.002	0.002	
IMAT [cm^2^]			
T0	7.2 ± 4.8 [5.8–8.7]	6.7 ± 4.5 [5.3–8.2]	0.642
T1	11.8 ± 7.1 [9.7–14.0]	10.6 ± 6.5 [8.4–12.7]	0.401
Mean difference	4.6 ± 4.9 [3.1–6.1]	3.8 ± 4.1 [2.4–5.2]	
*p*-value	<0.001	<0.001	
%IMAT			
T0	6.2 ± 3.4 [5.2–7.3]	8.1 ± 4.1 [6.7–9.4]	0.028
T1	9.3 ± 4.1 [8.0–10.5]	11.4 ± 4.4 [10.0–12.9]	0.025
Mean difference	3.0 ± 2.9 [2.2–3.9]	3.4 ± 3.2 [2.3–4.5]	
*p*-value	<0.001	<0.001	
LAMA [cm^2^]			
T0	30.5 ± 10.0 [27.4–33.6]	21.9 ± 6.5 [19.7–24.1]	<0.001
T1	35.9 ± 9.8 [32.9–38.9]	25.6 ± 6.7 [23.3–27.8]	<0.001
Mean difference	5.4 ± 6.1 [3.5–7.3]	3.7 ± 5.4 [1.9–5.4]	
*p*-value	<0.001	<0.001	
%LAMA			
T0	29.1 ± 6.8 [27.0–31.2]	30.0 ± 6.4 [27.9–32.1]	0.542
T1	32.5 ± 4.9 [31.0–34.0]	33.0 ± 5.8 [31.1–35.0]	0.637
Mean difference	3.4 ± 4.9 [1.9–4.9]	3.1 ± 6.2 [1.0–5.1]	
*p*-value	<0.001	0.005	
IMAT/SMA ratio			
T0	6.8 ± 4.0 [5.5–8.0]	9.0 ± 5.0 [7.3–10.7]	0.032
T1	10.4 ± 5.2 [8.8–12.0]	13.2 ± 5.9 [11.2–15.2]	0.028
Mean difference	3.7 ± 3.6 [2.6–4.8]	4.2 ± 4.0 [2.9 ± 5.6]	
*p*-value	<0.001	<0.001	
skeletal muscle indices (SMI)			
SMA/height [m^2^]			
T0	32.7 ± 5.6 [31.0–34.5]	26.8 ± 4.1 [25.4–28.1]	<0.001
T1	34.7 ± 6.2 [32.8–36.6]	28.4 ± 4.6 [26.9–29.9]	<0.001
Mean difference	2.0 ± 3.6 [0.9–3.1]	1.6 ± 2.9 [0.7–2.6]	
*p*-value	<0.001	0.004	
SMA/kg			
T0	1.5 ± 0.2 [1.5–1.6]	1.4 ± 0.1 [1.3–1.4]	<0.001
T1	1.5 ± 0.2 [1.4–1.6]	1.3 ± 0.1 [1.3–1.4]	<0.001
Mean difference	0 ± 0.1 [−0.1–0]	0 ± 0.1 [−0.1–0]	
*p*-value	0.054	0.015	
SMA/BMI [kg/m^2^]			
T0	4.8 ± 0.5 [4.7–4.9]	3.7 ± 0.4 [3.6–3.8]	<0.001
T1	4.7 ± 0.5 [4.6–4.9]	3.6 ± 0.4 [3.5–3.7]	<0.001
Mean difference	−0.1 ± 0.3 [−0.2–0.02]	−0.09 ± 0.2 [−0.2–−0.02]	
*p*-value	0.096	0.017	

Values are expressed as the mean ± standard deviation. Brackets indicate the 95% confidence intervals (CI). ETI, elexacaftor, tezacaftor, ivacaftor; ppFEV1, percent predicted forced expiratory volume in 1 s; BMI, body mass index; IMAT, inter- and intramuscular adipose tissue (−30 to −190 HU); LAMA, low-attenuation muscle area (−29 to 29 HU); SMA, skeletal muscle area.

## Data Availability

The original contributions presented in this study are included in the article/Supplementary Material. Further inquiries can be directed to the corresponding author.

## Supplementary Materials

The following supporting information can be downloaded at: https://www.mdpi.com/article/10.3390/medsci13040284/s1, Table S1: strobe checklist.

## Abbreviations

BMI	body mass index
CT	computed tomography
ETI	elexacaftor/tezacaftor/ivacaftor
pwCF	people with cystic fibrosis
IMAT	inter- and intramuscular adipose tissue
SMA	skeletal muscle area
SMI	skeletal muscle indices
LAMA	low-attenuation muscle area
ppFEV1	percent predicted forced expiratory volume in 1 s
